# Arbuscular mycorrhizal fungi alleviate Mn phytotoxicity by altering Mn subcellular distribution and chemical forms in *Lespedeza davidii*

**DOI:** 10.3389/fpls.2024.1470063

**Published:** 2024-11-27

**Authors:** Gao Pan, Jiayao Hu, Zhen Zi, Wenying Wang, Xinhang Li, Xiaoli Xu, Wensheng Liu

**Affiliations:** ^1^ College of Life and Environmental Sciences, Central South University of Forestry and Technology, Changsha, China; ^2^ Hunan Research Center of Engineering Technology for Utilization of Environmental and Resources Plant, Central South University of Forestry and Technology, Changsha, China

**Keywords:** arbuscular mycorrhizal fungi, manganese stress, *Lespedeza davidii*, physiological characteristics, subcellular distribution, chemical forms

## Abstract

**Introduction:**

Arbuscular mycorrhizal fungi (AMF) can relieve manganese (Mn) phytotoxicity and promote plant growth under Mn stress, but their roles remain unclear.

**Methods:**

In this study, *Lespedeza davidii* inoculated with or without AMF (*Glomus mosseae*) under different Mn concentrations (0 mmol/L, 1 mmol/L, 5 mmol/L, 10 mmol/L, and 20 mmol/L) was cultivated via a pot experiment, and plant biomass, physiological and biochemical characteristics, manganese absorption, subcellular distribution, and chemical forms of Mn were examined.

**Results:**

The results showed that root biomass, stem biomass, leaf biomass, and total individual biomass decreased under high Mn concentrations (above 10 mmol/L), and the inoculated plants had higher biomass than the uninoculated plants. With the increasing Mn concentration, the contents of soluble sugar, soluble protein, free proline, superoxide dismutase (SOD), peroxidase (POD), and catalase (CAT) increased first and then decreased, while the malondialdehyde (MDA) content increased. The contents of soluble sugar, soluble protein, free proline, SOD, POD, and CAT in the inoculated group were higher than those in the uninoculated group at the Mn concentration of 20 mmol/L. The content of MDA in the inoculated plants was lower than that in the uninoculated plants. AMF inoculation enriched most of the manganese in the root system when compared with the non-mycorrhizal treatment. Subcellular distribution of Mn indicated that most of the Mn ions were stored in the cell wall and the vacuoles (the soluble fractions), and the proportion of Mn content in the cell wall components and the vacuole components in leaves in the inoculated group was higher than that in the uninoculated group. Furthermore, the proportions of Mn extracted using ethanol and deionized water in the uninoculated group in stems and roots were higher than those in the inoculated group, which suggested that AMF could convert Mn into inactive forms.

**Discussion:**

The present study demonstrated that AMF could improve the resistance of *L. davidii* to Mn toxicity by increasing the activity of antioxidant enzymes and altering the subcellular distribution and chemical forms of Mn.

## 1 Introduction

Manganese (Mn) is an important essential trace element for plants, as it is involved in plant photosynthesis, enzymatic reactions, and redox activities. However, high concentrations of Mn would lead to destructed chloroplast structure, reduced plant photosynthesis, and inhibited growth for plants (Bai et al., 2021; Lin et al., 2021). They would even harm human health through the food chain (Santos et al., 2017; Luo et al., 2020). However, Mn is also one of the most widely used heavy metals in industry, manganese ore has been exploited for a long time, and large areas of manganese mine wasteland bring heavy Mn pollution. This seriously influences the normal production and living of people in these areas. Therefore, it is urgent to control Mn pollution in these areas as soon as possible.

Of the heavy metal (HM) treatment methods, arbuscular mycorrhizal fungi (AMF)-favored phytoremediation is the most economical and persistent technology with less secondary pollutant effects, which has received much attention in recent years (Riaz et al., 2021). AMF are a kind of widespread soil microorganism, which can coexist with more than 80% of terrestrial plants. These microorganisms have a good symbiotic relationship with plants. They could contribute to increasing plant metal tolerance and improving plant growth performance (Amir et al., 2019; Marro et al., 2022). In addition, AMF can enhance the uptake of mineral nutrition and directly affect the absorption and accumulation of heavy metals in plants under heavy metal stress (Garcia et al., 2018; Boorboori and Zhang, 2022; Riaz et al., 2021). Moreover, AMF (such as *Glomus mosseae*) can widely exist in real-scale wasteland habitats around the world (e.g., USA, China, Mexico, Portugal, and Algeria) (Pan et al., 2023b). Therefore, AMF have a wide prospect for phytoremediation of contaminated soils (Wang et al., 2022).

Plant growth under stress reflects the adaptability of plants to the environment and their bioremediation capability. Appropriate amounts of Mn would contribute to plant growth and development, while excessive amounts of Mn would markedly impair plant growth and reduce plant biomass (Xiao et al., 2020). In addition, Mn accumulation within plants plays a key role in plant survival under stress. Higher concentrations of Mn would easily accumulate in plants from soils and cause damage. AMF could promote the growth of host plants by reducing heavy metal concentrations in shoots and roots (Zhang et al., 2019; Pan et al., 2023a). Understanding the enrichment characteristics of Mn is key to revealing the adaptive mechanism.

In response to high amounts of Mn, plants produce an excessive quantity of reactive oxygen species (ROS), which may result in the malfunctioning of plant cells (Han et al., 2021; Kaya et al., 2022). To prevent the cells from oxidative damage, the defense proteins [e.g., superoxide dismutase (SOD), peroxidase (POD), and catalase (CAT)] would enhance the capacity of plants to combat excessive ROS. AMF could demonstrate high activities of antioxidant enzymes to alleviate oxidative stress and decrease the contents of malondialdehyde (MDA) (Wang et al., 2021a). In addition to the enzymatic defense mechanisms, osmoregulation substances, such as soluble sugar, soluble protein, and free proline, would also be produced to maintain the water balance and energy supply in cells and to perform the protective function.

Subcellular distribution and chemical forms of heavy metal in plants are two internal mechanisms for plants that impart protection against heavy metal toxicity (Wang et al., 2021b). Chelation and compartmentalization play essential roles in alleviating Mn toxicity in plants. Deposition in the cell wall and vacuolar (the soluble fraction) sequestration could prevent HM from entering more vulnerable sites in cells. In addition, Mn would distribute in different chemical forms once taken up by plants. Plants could reduce Mn migration and toxicity by converting highly toxic chemical forms of Mn into less toxic chemical forms in plants. Therefore, investigating the subcellular distribution and chemical forms of Mn in plants was a key step to revealing the role of AMF in plants under stress.

*Lespedeza davidii* is a shrub of *Lespedeza* in Fabaceae, which is widely distributed in southern China (Zheng et al., 2011b). This plant has a strong ability for biological nitrogen fixation and soil and water conservation (Wang et al., 2021b; Rai et al., 2021). It was reported that the species of Fabaceae could decrease the injury of heavy metals in soybean rhizobia (Doty, 2008). As a Fabaceae plant, *L. davidii* has strong heavy metal resistance. It can grow normally in a variety of habitats. The investigation on the abandoned manganese tailings of Xiangtan, Hunan Province, showed that *L. davidii* grows well and can normally blossom and bear fruits in this area, whose soil Mn concentration reached 52,319 mg/kg. It indicated that this plant has a strong tolerance to Mn pollution (Hu et al., 2022). Therefore, the plant is an important restoration candidate for the manganese mine area. At present, relevant research on *L. davidii* mainly focuses on its medicinal value (Li and Wang, 1989; Liang and Yan, 2018) and tolerance to Pb stress (Zheng et al., 2011a). The adaptive mechanism of this plant inoculated with AMF under Mn stress has not been systematically studied, which limited the further application of the plant.

In the present study, seedlings of *L. davidii* were cultivated under different Mn concentrations; whether or not to inoculate with AMF, plant growth, physiological and biochemical characteristics, enrichment characteristics, subcellular components, and chemical morphology of Mn were measured. The aim of this study is 1) to determine whether AMF can affect the plant growth of *L. davidii* under Mn stress, 2) to reveal the physiological mechanism of *L. davidii* to Mn stress under the condition of AMF inoculation, and 3) to illustrate whether AMF can regulate the migration and distribution of Mn and its chemical state transformation, and if so, what the regulatory mechanism is.

## 2 Materials and methods

### 2.1 Material provision

Seeds of *L. davidii* were collected in Xiangtan manganese tailings, Hunan Province, in November 2019 (28°03′E, 112°55′N). The field investigation revealed that the height of *L. davidii* was approximately 1.4 m. It also showed that the plant had set many flowers and bear fruits. In the present study, over 80 well-growing individuals of *L. davidii* were selected, and their mature and plump fruits were sampled and brought back to the laboratory in cloth bags. The seeds (picked from fruits) were stored at room temperature until seed germination. The average 1000 seed weight of this plant was 9.39 g.

The AMF species [*G. mosseae* (GM)], which was highly resistant to Mn, was obtained from the Institute of Plant Nutrition, Resources and Environment, Beijing Academy of Agriculture and Forestry Sciences, China (Pan et al., 2023a). The fungi were propagated using maize as a host plant, and the mycelium, spores, colonized root fragments, and dried sand–soil were mixed to use as AMF inoculant according to Yang et al. (2014). Average spore densities were 590 per 10 g of air-dried soil.

### 2.2 Pot experiment

The seed germination and seedling cultivation of *L. davidii* were carried out in the greenhouse of Central Southern University of Forestry and Technology in August 2021. After the seedlings were cultivated for 0.5 years, the uniform 18-cm-tall seedlings of *L. davidii* were chosen and transferred to the pot with a height of 11 cm and a diameter of 18 cm, which was sterilized with 75% ethanol solution. Each pot was filled with 2 kg of sterilized sand mix (pure sand:perlite = 2:1), which was sterilized with high-pressure steam at 121°C for 2 h.

The pot experiment was performed in a completely randomized design with a two factorial scheme with the following variables: five Mn-added levels (0, 1, 5, 10, and 20 mg Mn kg^−1^ soil) (Pan et al., 2023a), two treatments (inoculation with or without GM), and 10 replicates in each treatment, for a total of 100 pots.

The GM inocula were applied at a rate of 5% (w/w) of the weight. The same amounts of the autoclaved inocula were added to uninoculated controls and supplemented with a filtrate obtained from the unsterilized soil solution sieved using an 11-μm sieve to provide the microbial populations accompanying the mycorrhizal fungi (Zhong et al., 2012). The Mn concentration was determined by MnCl_2_·4H_2_O, and the same amount of distilled water was set as the control group (CK). The transplanted plants were cultured in the greenhouse (25°C–28°C) for 30 days.

During the growing period, Hoagland nutrient solution was added every 3 days. After 60 days of Mn stress, the plant growth traits, physiological and biochemical indices, Mn content, Mn subcellular components, and chemical forms of *L. davidii* were determined after harvesting.

### 2.3 Morphological measurements

Plant biomass was measured following plant harvest, and the individual plants were divided into three parts (root, stem, and leaf). All parts were dried for 2 h at 105°C followed by 48 h at 70°C to achieve a constant weight. The biomass of the roots, stems, and leaves of individual plants was determined using an electronic balance.

### 2.4 Mycorrhizal colonization measurements

To ensure that our inoculation protocol was successful, 60-day-old roots were checked using a combination of bright-field and confocal microscopy (Leica Microsystems GmbH, Wetzlar, Germany). The fresh roots were cut into fragments approximately 1 cm and then stained with 0.05% Trypan Blue according to the method of Phillips and Hayman (1970). Bright-field microscopy was used to assess mycorrhizal infection rate after the Trypan Blue staining (Phillips and Hayman, 1970). The infection rate was determined based on the microstructure of the infected *L. davidii* roots by identifying the internal hyphae, external hyphae, arbuscules, and spores. The AMF infection rate (ratio of infected roots) was quantified on 1-cm-long root segments using the grid-line intersect method under a compound microscope (40 × 10) (Giovannetti and Mosse, 1980). The results showed that the root colonization rates of plants inoculated with GM were 73% ± 2.9%.

### 2.5 Determination of osmoregulation substances and antioxidant systems

A leaf sample of approximately 0.2 g was ground using a mortar with liquid nitrogen, which was used to determine the physiological and biochemical indices. The activity of SOD was determined using the nitro blue tetrazolium (NBT) method. The amounts of soluble sugar content, free proline content, POD, CAT, and MDA were determined according to the methods of Gao (2000). Soluble protein content was determined using the method of Bradford (1976) with bovine serum albumin (BSA) as a standard. All the measurements were carried out in five replicates.

### 2.6 Mn concentration analysis

Mn concentration was determined according to the methods of Xiao et al. (2020). The dried roots, stems, and leaves were ground using a grinder, and the fine powder was weighed and placed in a muffle furnace at 300°C for 2 h followed by 500°C for 5 h. Then, the powders were soaked in concentrated HNO_3_:HClO_4_ (7:3, v/v) using the Kjeldahl method until the samples became a clear liquid (Rao et al., 2011). The samples were diluted to a constant volume (25 mL), and the Mn concentrations were determined using inductively coupled plasma–optical emission spectroscopy (ICP-OES) (iCAP6500, Thermo Scientific, Oxford, UK). Measurements of the plant’s Mn concentrations were performed in three replicates (Xiao et al., 2020). The bioconcentration factors (BCFs) were defined as the ratio of metal concentration in plant roots to that in the soil. The transport coefficient (TF) was indicated as the ratio of metal concentration in plant shoots to that in the roots.

### 2.7 Subcellular fraction extraction

To reveal the effects of various treatments on the distribution of Mn across different subcellular fractions, the concentrations of Mn with different subcellular compartments were determined using the gradient centrifugation technique, and cells were separated into four fractions [cell wall fraction, chloroplast and cell nuclear fraction, mitochondrial fraction, and ribosomal fraction (soluble components)] (Wang et al., 2015). Frozen plant materials were homogenized in cooled extraction buffer [50 mM Tris–HCl, 250 mM sucrose, and 1.0 mM dithioerythritol (C_4_H_10_O_2_S_2_), pH 7.5] using a chilled mortar and pestle. The homogenate was sieved through nylon cloth (80 μm), and the liquid was squeezed from the residue. The residue was washed twice with homogenization buffer; as it contained mainly cell walls and cell wall debris, it was designated as the “cell wall fraction”. Then, the supernatant was moved to a new centrifuge tube and centrifuged at 2,000 ×*g* for 15 min, creating a pellet consisting of chloroplasts and cell nuclei. The supernatant was once again removed and centrifuged at 10,000 ×*g* for 20 min, creating a pellet that was considered the mitochondrial fraction. In the end, the final supernatant was considered the ribosomal fraction (including vacuoles). All steps were performed at 4°C, and the subcellular fractions were dried at 70°C to a constant weight (Wang et al., 2015).

### 2.8 Chemical form extraction

Chemical forms of Mn were extracted in the following order: 1) 80% ethanol, extracting inorganic Mn giving priority to nitrate/nitrite, chloride, and aminophenol manganese; 2) deionized water (d-H_2_O), extracting water soluble Mn–organic acid complexes; 3) 1 M NaCl, extracting pectates and protein integrated Mn; 4) 2% acetic acid (HAc), extracting undissolved manganese phosphate; 5) 0.6 M HCl, extracting manganese oxalate; and 6) the residues (Fu et al., 2011).

Frozen tissues were homogenized in an extraction solution using a mortar and pestle, diluted at a ratio of 1:100 (w/v), and shaken for 22 h at 25°C. The homogenate was then centrifuged at 5,000 ×*g* for 10 min, obtaining the first supernatant solution in a conical beaker. The sedimentation was re-suspended twice in an extraction solution, shaken for 2 h at 25°C, and centrifuged at 5,000 ×*g* for 10 min. The supernatants of the three suspensions were then pooled. The residue was extracted with the next extraction solution in the sequence using the same procedure described above. Each pooled solution was evaporated on an electric plate at 70°C to a constant weight.

### 2.9 Statistical analysis

All results were presented as means ± standard deviation (SD) for the 10 replicates. Two-way ANOVA was used to test the data of *L. davidii* between different Mn concentration treatment groups and inoculation conditions, and Duncan’s multiple comparison method (the significance level was set as 0.05) was used to conduct significant difference analysis between different concentrations. Statistical analyses were conducted using the SPSS 22.0 software package (SPSS, Inc., Chicago, IL, USA), and figures were created using SigmaPlot 14.0.

## 3 Results

### 3.1 Plant biomass

**Figure 1:**
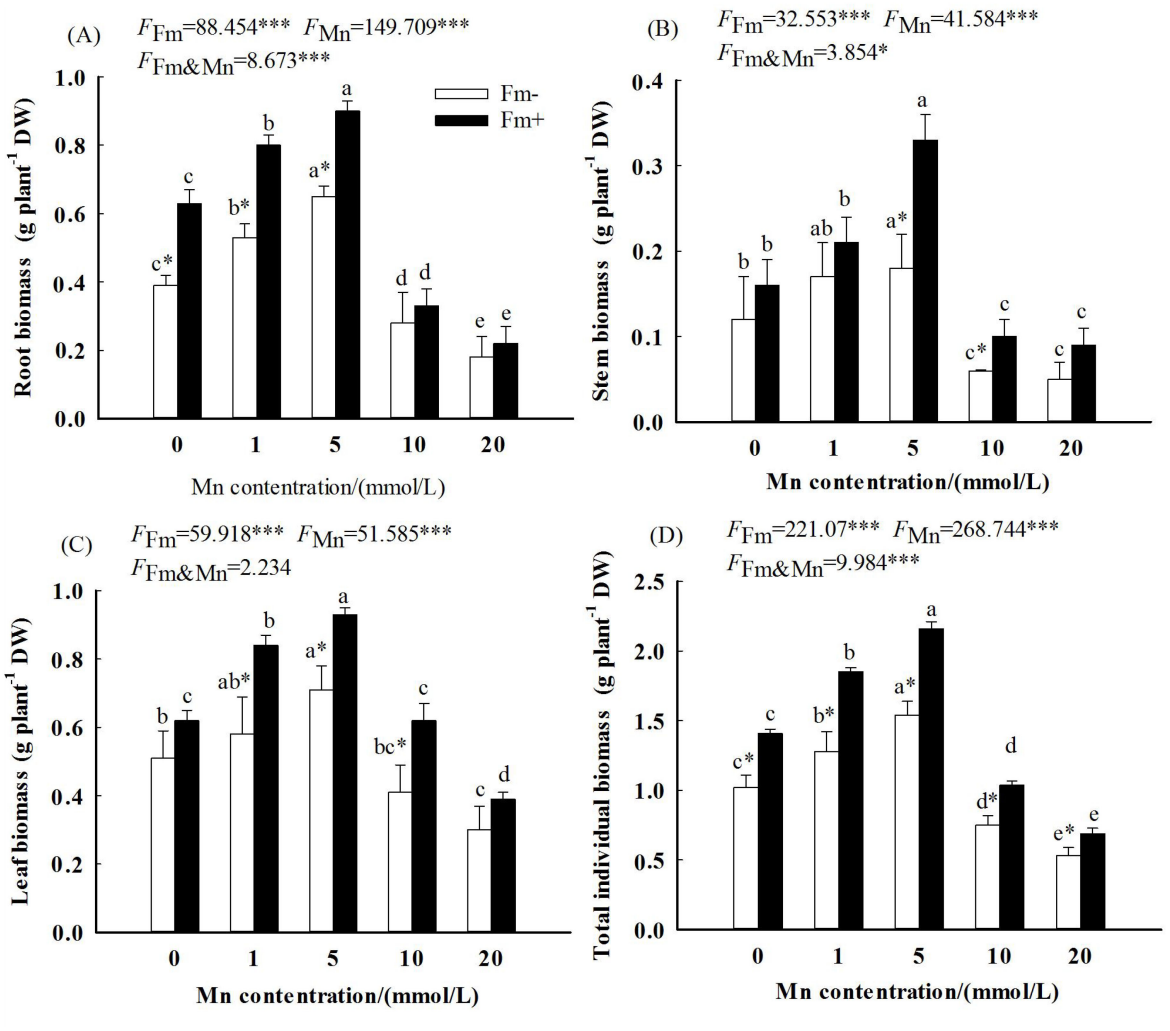
Effects of Mn treatments on stem biomass **(A)**, root biomass **(B)**, leaf biomass **(C)**, and total individual biomass **(D)** of *Lespedeza davidii* plants with AMF inoculation (Fm+) and non-inoculation (Fm−). All data show the means ± SE of 10 replicates. Values with different small letters within the same column indicate significant differences at the *p* < 0.05 level between Mn treatments. ^*^ indicates significant difference (*p* < 0.05) between AMF-inoculated plants and uninoculated plants. AMF, arbuscular mycorrhizal fungi. *** indicates highly significant difference (p<0.001).

The two-way ANOVA showed significant and interactive effects of Mn stress and AMF inoculation on plant biomass; all the root biomass, stem biomass, leaf biomass, and total individual biomass of *L. davidii* were significantly affected by Mn concentrations and AMF inoculation (*p* < 0.001). All the root biomass, stem biomass, leaf biomass, and total individual biomass of plants in the AMF-inoculated and uninoculated plants increased first and then decreased (**Figures 1A–D**). Compared with the uninoculated plants, the root biomass (**Figure 1A**), stem biomass (**Figure 1B**), leaf biomass (**Figure 1C**), and total individual biomass (**Figure 1D**) of the AMF-inoculated plants were higher. The results showed that the positive effects of AMF on plant biomass compensated for the negative effect of Mn application.

### 3.2 Physiological characteristics

**Figure 2:**
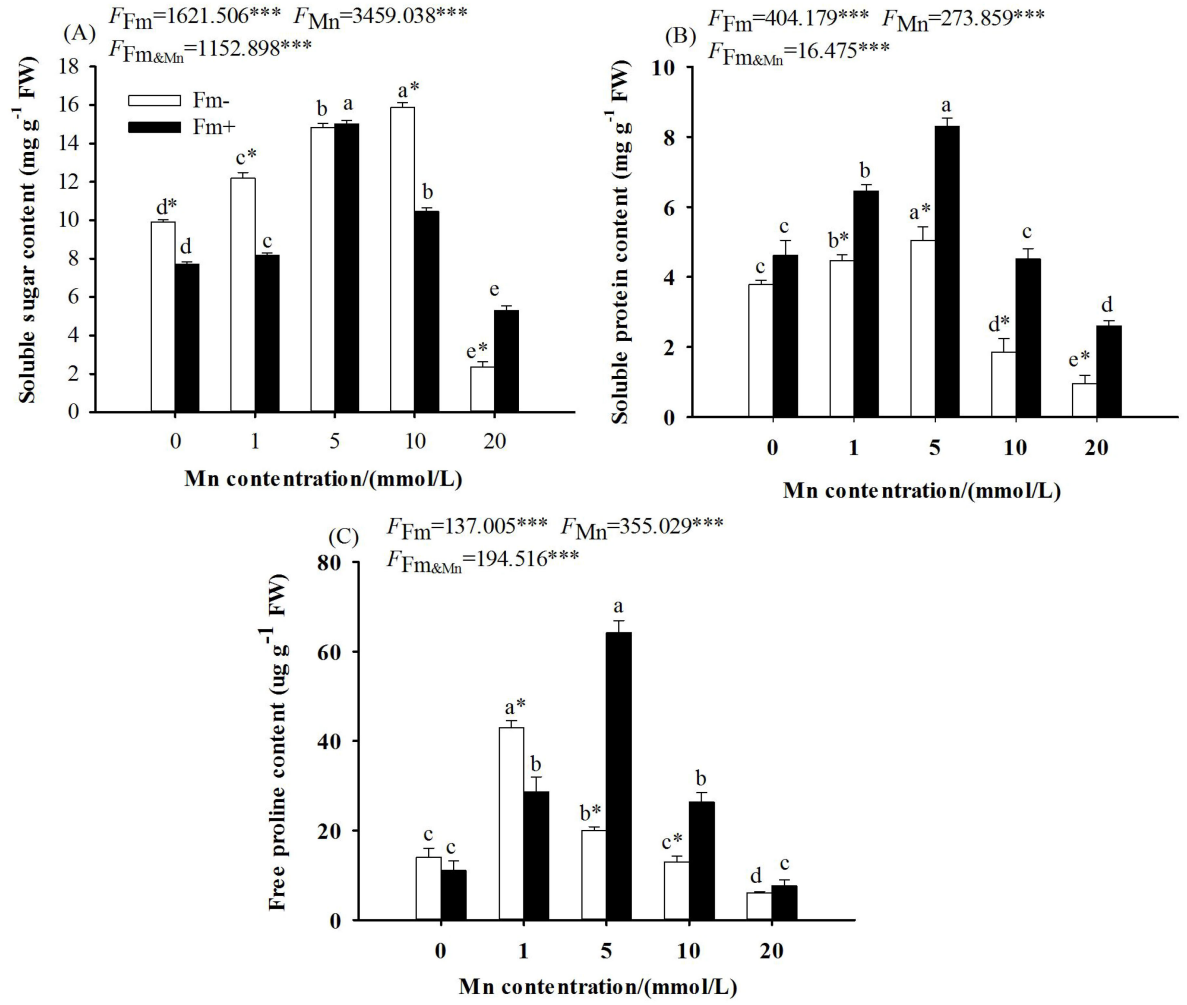
Effects of Mn treatments on the content of soluble sugar **(A)**, soluble protein **(B)**, and free proline **(C)** of *Lespedeza davidii* plants with AMF inoculation (Fm+) and non-inoculation (Fm−). Values with different small letters within the same column indicate significant differences at the *p* < 0.05 level between Mn treatments. ^*^ indicates significant difference (*p* < 0.05) between AMF-inoculated plants and uninoculated plants. AMF, arbuscular mycorrhizal fungi. *** indicates highly significant difference (p<0.001).

The two-way ANOVA showed that Mn stress and whether or not to inoculate with AMF had significant effects on the contents of soluble sugar, soluble protein, and free proline in the leaves of *L. davidii* (**Figure 2**; *p* < 0.001). With the increasing Mn concentration, the contents of soluble sugar (**Figure 2A**), soluble protein (**Figure 2B**), and free proline (**Figure 2C**) in *L. davidii* in the inoculated group and the uninoculated group increased first and then decreased. The contents of soluble sugar (**Figure 2A**), soluble protein (**Figure 2B**), and free proline (**Figure 2C**) in the inoculated group were higher than those in the uninoculated group at the Mn concentration of 20 mmol/L, and the values of the inoculated group were respectively 2.25, 2.70, and 1.26 times those of the uninoculated group.

**Figure 3:**
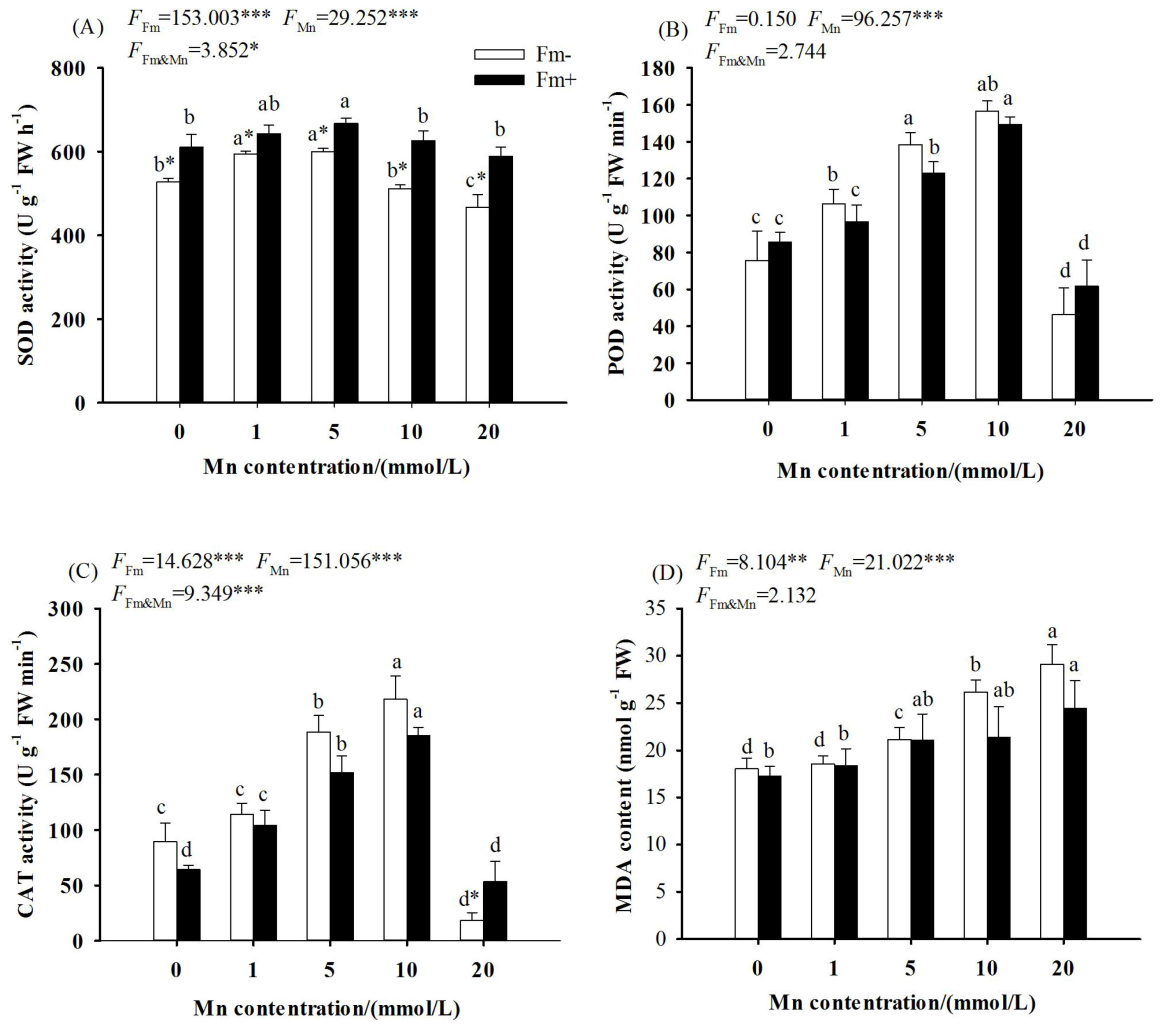
Effects of Mn treatments on superoxide dismutase (SOD; **A**), peroxidase (POD; **B**), catalase (CAT; **C**), and malondialdehyde (MDA; **D**) of *Lespedeza davidii* plants with AMF inoculation (Fm+) and non-inoculation (Fm−). Values with different small letters within the same column indicate significant differences at the *p* < 0.05 level between Mn treatments. ^*^ indicates significant difference (*p* < 0.05) between AMF-inoculated plants and uninoculated plants. AMF, arbuscular mycorrhizal fungi. ** indicates highly significant difference (p<0.01); *** indicates highly significant difference (p<0.001).

The two-way ANOVA showed that the effects of Mn stress and AMF inoculation on SOD, POD, and CAT activities reached a significant level (*p* < 0.001), the interaction between Mn stress and AMF inoculation on the effects on SOD activities reached a significant level (*p* < 0.05), and the effects on CAT activities reached a very significant level (*p* < 0.001), while the effects on POD activities did not reach a significant level. As the Mn concentration increased, the activities of SOD (**Figure 3A**), POD (**Figure 3B**), and CAT (**Figure 3C**) in the inoculated group and the uninoculated group increased first and then decreased with the increase of Mn concentration, and all decreased to the lowest values at the manganese concentration of 20 mmol/L (*p* < 0.001). The activities of SOD, POD, and CAT in the inoculated group were higher than those in the uninoculated group at the Mn concentration of 20 mmol/L. When the concentration of Mn was 20 mmol/L, the activities of SOD, POD, and CAT in the inoculated group were respectively 1.26, 1.33, and 2.94 times those of the uninoculated group.

The two-way ANOVA showed that the effects of Mn stress and whether or not to inoculate with AMF on the MDA content in *L. davidii* leaves reached a significant level (*p* < 0.001), while the interaction between Mn stress and AMF inoculation did not reach a significant level. With the increasing Mn concentration, the MDA content in *L. davidii* in the inoculated group and the uninoculated group increased (**Figure 3D**). When the Mn concentration was 20 mmol/L, the content of MDA in the inoculated group and the uninoculated group increased by 41.39% and 61.17%, respectively, compared with the control (**Figure 3D**; *p* < 0.001).

### 3.3 Mn accumulation

**Figure 4:**
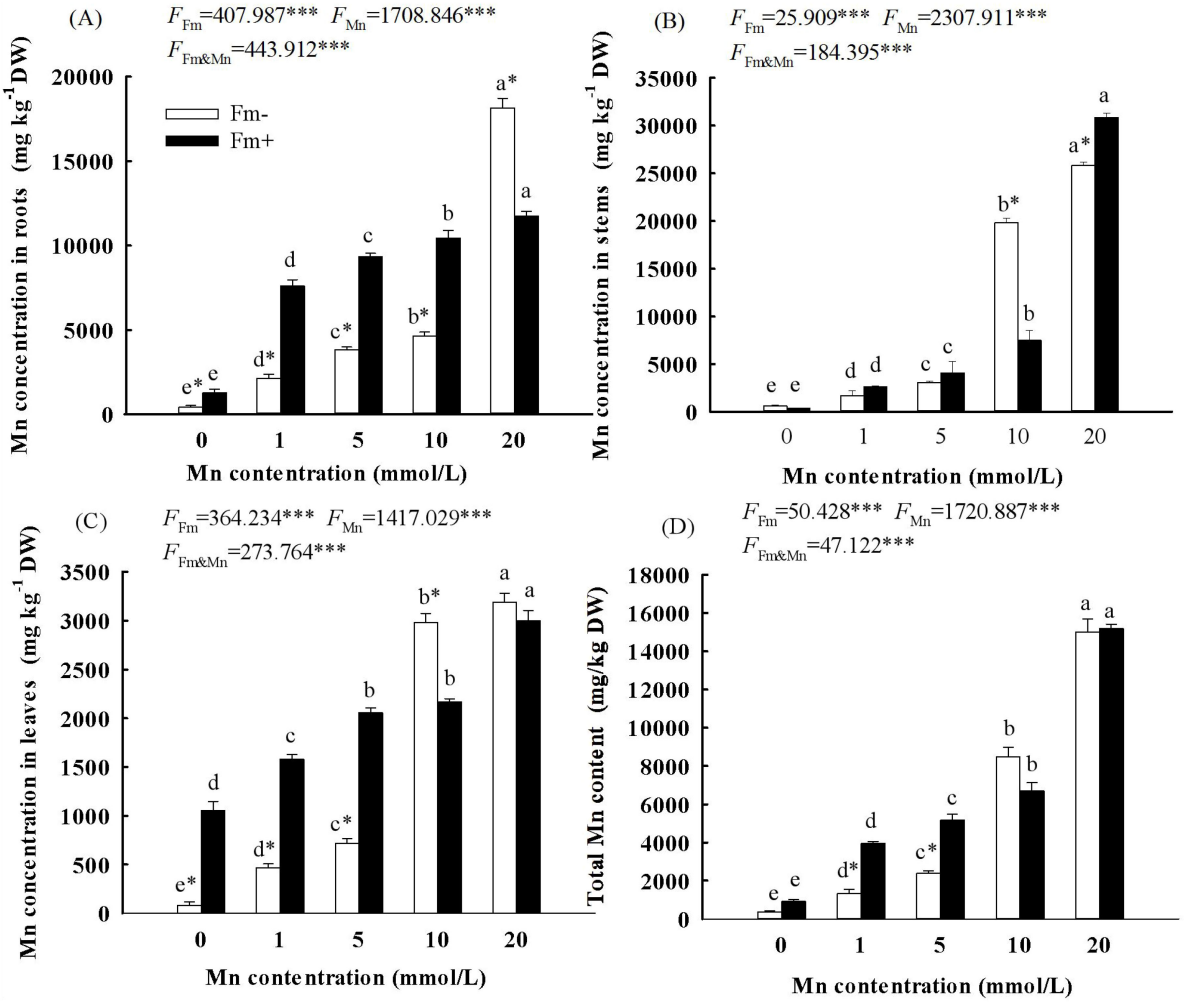
Effects of Mn treatments on the content of stems **(A)**, roots **(B)**, leaves **(C)**, and total individual **(D)** of *Lespedeza davidii* plants with AMF inoculation (Fm+) and non-inoculation (Fm−). Values with different small letters within the same column indicate significant differences at the *p* < 0.05 level between Mn treatments. ^*^ indicates significant difference (*p* < 0.05) between uninoculated plants and AMF-inoculated plants. AMF, arbuscular mycorrhizal fungi. *** indicates highly significant difference (p<0.001).

With the increasing Mn concentration, the Mn content of the roots (**Figure 4A**), stems (**Figure 4B**), leaves (**Figure 4C**), and total individual (**Figure 4D**) in the inoculated group and the uninoculated group increased. The two-way ANOVA showed that both Mn concentration and whether or not to inoculate with AMF had a significant effect on the Mn accumulation of the roots, stems, leaves, and total individual in *L. davidii* (**Figure 4**; *p* < 0.001). When the Mn concentration reached 20 mmol/L, the total Mn content in *L. davidii* in the inoculated group and the uninoculated group reached 15,199.81 mg/kg and 15,002.30 mg/kg, respectively. The content of Mn in the leaves of the inoculated group was lower than that of the uninoculated group when the concentration of Mn was 10 mmol/L and 20 mmol/L (**Figure 4C**; *p* < 0.05).

The two-way ANOVA showed that both Mn concentration and whether or not to inoculate with AMF had a significant effect on the BCF and TF of *L. davidii* (**Table 1**; *p* < 0.001). With the increasing Mn concentration, the BCF in both the inoculated and uninoculated groups decreased. At different Mn concentrations, the BCF of the inoculated group was higher than that of the uninoculated group.

**Table 1:** Effect of AMF on Mn bioconcentration coefficients (BCFs) and transport coefficients (TFs) of *Lespedeza davidii* leaves under Mn stress.

Mn concentration (mmol/L)	Bioconcentration coefficients/BCF		Transport coefficients/TF	
	+AMF	−AMF	+AMF	−AMF
CK			1.15 ± 0.20b	0.79 ± 0.15b
1	95.75 ± 2.78b*	32.06 ± 5.28b	0.56 ± 0.03d	0.50 ± 0.13c
5	25.01 ± 1.56b*	11.56 ± 0.58b	0.66 ± 0.14d	0.49 ± 0.02c
10	16.25 ± 1.05b*	20.56 ± 1.20b	0.92 ± 0.09c*	2.45 ± 0.08a
20	18.42 ± 0.26b	18.18 ± 0.83b	2.88 ± 0.05a*	0.80 ± 0.02b
*F*_AMF_	78.926***		32.047***	
*F*_Mn_	320.277***		189.731***	
*F*_Mn×AMF_	63.636***		206.837***	

^*^ indicates significant difference (*p* < 0.05) between uninoculated plants and AMF-inoculated plants; ** indicates highly significant difference (p<0.01); *** indicates highly significant difference (p<0.001).Values with different small letters within the same column indicate significant differences at the p < 0.05 levelbetween Mn treatments.

With the increase of Mn concentration, the TF of the inoculated group increased, while that of the uninoculated group increased first and then decreased (**Table 1**; *p* < 0.05). At different concentrations, the TF of the inoculated group was higher than that of the uninoculated group.

### 3.4 Subcellular components of Mn

**Figure 5:**
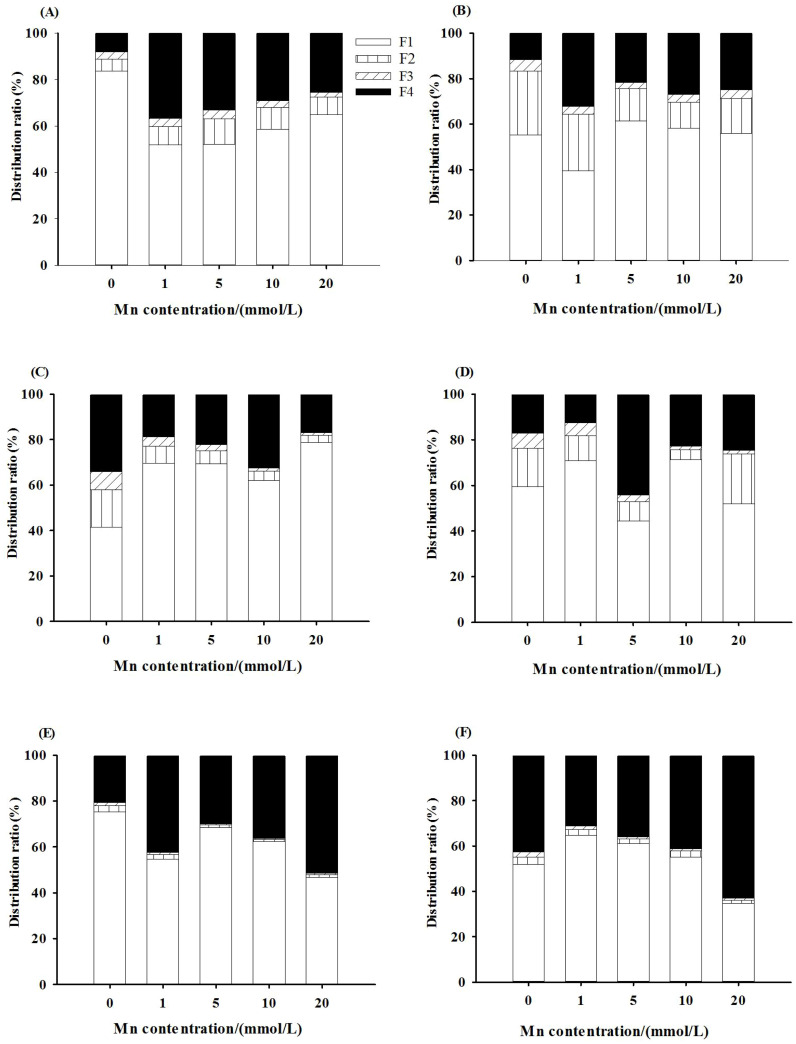
Manganese subcellular fraction distribution ratio of roots **(A, B)**, stems **(C, D)**, and leaves **(E, F)** of *Lespedeza davidii* in inoculated group and uninoculated group under Mn stress. **(A, C, E)** Uninoculated group. **(B, D, F)** Inoculated group. F1, cell wall fraction; F2, chloroplast and cell nuclear fraction; F3, mitochondrial fraction; F4, ribosomal fraction.

The two-way ANOVA showed that both Mn concentration and whether or not to inoculate with AMF had significant effects on the content of Mn in the cell wall components, soluble components (ribosomal fraction), mitochondrial components, and chloroplast and nuclear components of the roots, stems, and leaves of *L. davidii* (*p* < 0.001). With the increase of Mn concentration, the content of Mn in the cell wall components, chloroplast and nuclear components, mitochondrial components, and soluble components of the roots, stems, and leaves of *L. davidii* in the inoculated and uninoculated groups increased (*p* < 0.05). The Mn accumulations and distribution proportions in different subcellular fractions of the roots, stems, and leaves are presented in **Figure 5** and **Supplementary Figures S1**–**S3**, which showed that a large percentage of Mn (80%–90%) was accumulated in cell walls and the soluble fractions in the roots (**Figures 5A, B**), stems (**Figures 5C, D**), and leaves (**Figures 5E, F**), and a small portion of Mn presented in the cellular organelles (the chloroplast and nuclear components and mitochondrial components). Compared with that of the uninoculated group, the proportion of Mn content in the cell wall components and the soluble components in the leaves of the inoculated group was higher (**Figures 5E, F**).

### 3.5 Chemical forms of Mn

**Figure 6:**
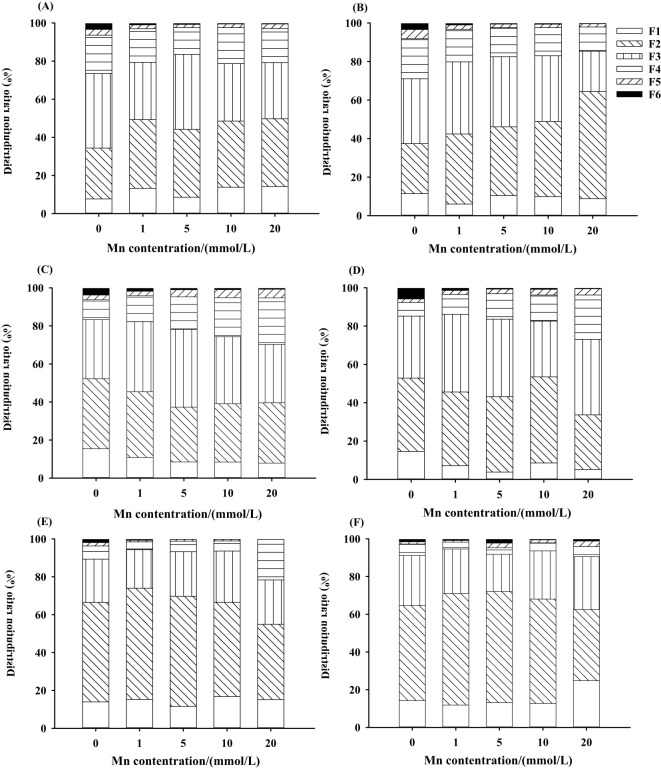
Manganese chemical form distribution ratio of roots **(A, B)**, stems **(C, D)**, and leaves **(E, F)** of *Lespedeza davidii* in inoculated group and uninoculated group under Mn stress. **(A, C, E)** Uninoculated group. **(B, D, F)** Inoculated group. F1, 80% ethanol; F2, deionized H_2_O; F3, 1 M NaCl; F4, 2% HA; F5, 0.6 M HCl; F6, residue.

The effects of different treatments on the concentrations and proportions of Mn in different chemical forms in *L. davidii* are shown in **Figure 6** and **Supplementary Figures S4**-**S6**. With the increasing Mn concentration, the contents of six chemical forms of Mn extracts in the roots (**Supplementary Figure S4**), stems (**Supplementary Figure S5**), and leaves (**Supplementary Figure S6**) of *L. davidii* in the uninoculated group and the inoculated group increased (*p* < 0.05). Chemical forms of Mn extracted using 80% ethanol (FE), deionized water (FW), and 1 M NaCl (FNaCl) were predominant (more than 90%) in both the uninoculated and inoculated plants (**Figures 6A–F**). The two-way ANOVA showed that the effects of Mn stress, inoculation and interaction of Mn stress, and inoculation on the contents of Mn ethanol extract, deionized water extract, sodium chloride extract, and acetic acid extract in the roots of *L. davidii* were extremely significant (*p* < 0.001). The proportions of Mn extracted using ethanol and deionized water in the uninoculated group in stems and roots were higher than those in the inoculated group (**Figures 6C–F**).

## 4 Discussion

### 4.1 The effects of AMF on plant biomass under Mn stress

Biomass variation under heavy metal stress is an important standard to determine plant tolerance and the phytoremediation potential for plants (Han et al., 2021). In this study, the biomass of leaves, stems, roots, and the total individual of *L. davidii* increased first and then decreased with the increasing Mn concentration. This is consistent with the results on *Cleome viscosa* (Xiao et al., 2020) and *Rhus chinensis* (Pan et al., 2023a) under Mn stress. It showed that low promotion and high suppression are common phenomena for Mn in the growth of plants.

This study also showed that the inoculated plants of *L. davidii* had higher biomass than the uninoculated plants (**Figure 1D**). Chaturvedia et al. (2018) and Pan et al. (2023a) also found that the AMF-inoculated plants had higher plant height and biomass than the uninoculated counterparts. The present study is consistent with these studies. The reason lies in that AMFs are known to have the ability to change enzyme activity and increase the ability of host plants to resist heavy metal stress (Chen et al., 2019). In our study, the results showed that the contents of soluble sugar, soluble protein, free proline, SOD, POD, and CAT in the inoculated group were higher than those in the uninoculated group at the Mn concentration of 20 mmol/L. These would promote the growth of plants. In addition, previous studies also suggested that AMF can chelate Mn, thereby reducing the bioavailability of Mn. All of these would help plant growth under stress.

### 4.2 The effects of AMF on physiological characteristics under Mn stress

Osmotic regulation is an important mechanism for plants to cope with heavy metal stress. In order to resist the adverse environment, plants would produce and accumulate a large number of osmoregulation substances (such as soluble sugar, soluble protein, and free proline) to maintain the water balance and energy supply in cells, thereby reducing the stress damage caused by osmotic water loss and maintaining the normal life metabolism activities of cells. The present study showed that the contents of soluble sugar, soluble protein, and free proline in *L. davidii* in the inoculated and uninoculated groups increased first and then decreased with the increasing Mn concentration (**Figure 3**). Kuang et al. (2022) also found that osmoregulation substances increased first and then decreased with the increasing Mn concentration in *Daucus carota*. The reason is that Mn stress can activate the osmotic regulation system in *L. davidii* and resist Mn toxicity through the accumulation of osmotic regulation substances. When the Mn concentration is too high, the osmotic adjustment ability of *L. davidii* is insufficient to resist the toxicity of high Mn, the water balance mechanism is destroyed, and the plant resistance is weakened. The present study also showed that compared with those in the inoculated group, the contents of soluble protein (**Figure 2B**) and free proline (**Figure 2C**) in the inoculated group were higher when the Mn concentrations were 5 mmol/L, 10 mmol/L, and 20 mmol/L. This is the same as that in *Lolium perenne* (Xu et al., 2014) and *Tamarix chinensis* (Han et al., 2021) under salt stress, which suggested that AMF inoculation can effectively increase the content of osmoregulation substances. The reason is that AMF can regulate the osmotic regulation system to stabilize the physiological environment in plants to adapt to stress. AMF could change enzyme activity and increase the ability of host plants to resist oxidative stress (Pan et al., 2023b).

Mn stress would result in the accumulation of ROS such as H_2_O_2_, O_2_, and MDA in the plant, which potentially leads to plasma membrane damage and a decrease in physiological activities related to membrane function. SOD, POD, and CAT are important components of plant antioxidant enzyme promotion systems. The synergistic effect of these three enzymes can effectively reduce the accumulation of ROS in plants and reduce the damage of stress to plants. In this study, the POD and CAT activities of *L. davidii* in the inoculated and uninoculated groups increased first and then decreased with the increase of Mn concentration. This showed that Mn stress can activate the antioxidant system of *L. davidii* and resist Mn toxicity by improving the activity of antioxidant enzymes. When the Mn concentration is too high, the antioxidant system of *L. davidii* may be destroyed, the activity of antioxidant enzymes is reduced, and the plant defense ability is weakened. Mn stress damages the cell membrane and destroys the original region of the metabolism of the protective enzyme system in the cell. Mn may also directly replace the trace elements in the activity of some enzymes to change or even destroy the enzyme activity. The present study also illustrated that when the Mn concentration was 20 mmol/L, the activities of SOD, POD, and CAT in the inoculated group were higher than those in the uninoculated group. This is the same as the results of marigold under cadmium stress (Liu et al., 2011), *Trifolium repens* in HM-contaminated soils (Azcón et al., 2009), and *Solanum lycopersicum* under salinization stress (Cao et al., 2022). This indicated that AMF inoculation can enhance the resistance of plants to high Mn toxicity by improving the activity of antioxidant enzymes and reducing lipid peroxidation, which would promote plant physiological metabolism and reduce the toxicity and damage of heavy metals to plants. Therefore, it appeared that it was an important mechanism for AMF to alleviate the damage of Mn by enhancing the activity of antioxidant enzymes and small molecular osmotic regulators.

MDA is the final product of membrane lipid peroxidation, which can measure the degree of damage to plant cell plasma membrane under stress. The level of MDA can reflect the degree of plant poisoning and also indicate the level of plant stress resistance. The present study showed that compared with that of the uninoculated group, the MDA content of the inoculated group was lower under the relevant Mn concentrations (**Figure 3D**). Similarly, AMF inoculation can effectively reduce the membrane lipid peroxidation of *Medicago sativa* under cadmium stress, reduce the MDA content in the host, and improve tolerance (Wang et al., 2022). This showed that excessive Mn makes the antioxidant enzyme system in the cells of *L. davidii* disordered, the balance between the production and elimination of ROS is broken, and the production of ROS in large quantities causes oxidative damage to plant cells, leading to a large MDA accumulation. AMF inoculation could weaken the damage of Mn toxicity to the leaf cells of *L. davidii* and make the physiological and biochemical environments in *L. davidii* relatively stable. Therefore, it appears that AMF can repair oxidative damage in membranes and maintain homeostasis in the internal environment.

### 4.3 The effects of AMF on Mn accumulation under Mn stress

The enrichment characteristics of heavy metals in plants usually include three indicators, namely, heavy metal accumulation, bioaccumulation coefficient, and transport coefficient, which are the main factors determining whether they are tolerant to Mn stress. The present study showed that with the increasing Mn concentration, the Mn content in the roots, stems, and leaves of *L. davidii* in the inoculated group and the uninoculated group increased. Compared with that of the uninoculated group, the content of Mn in the leaves of the inoculated group was lower when the concentrations of Mn were 5 mmol/L and 20 mmol/L (**Figure 4**, *p* < 0.05). This indicated that inoculation with AMF significantly increased the uptake of Mn in soil by *L. davidii* roots and decreased the transportation to leaves. Similarly, mycorrhizal colonization significantly enhanced the fixation of cadmium in the root system of *Lotus japonicus* (Chen et al., 2018), AMF inoculation significantly improved the absorption of cadmium in the soil by the root system of *Capsicum annuum* (Mou et al., 2021), and mycorrhizal infection increased the copper binding capacity of the cell wall of *M. sativa* (Wang et al., 2012). AMF inoculation often promotes the host plant and AMF to form a rhizobacterial structure. Plants can rely on this structure to expand the absorption range of nutrients and to improve the host’s absorption of nutrients in the soil under adverse environments (Wipf et al., 2019). As a nutrient element of plants, Mn absorption by AMF will also increase, which will increase the Mn content in *L. davidii*. Comparing the inoculated group with the uninoculated group, the BCF and TF of the inoculated group were generally higher than those of the uninoculated group. Similarly, AMF inoculation significantly increased the enrichment coefficient and reduced the transport of heavy metals from roots to aboveground parts for *C. annuum* (Mou et al., 2021). Research found that when the concentration of heavy metals in the soil is high, AMF can fix most of the heavy metals in the roots or fungal rhizosphere of plants and inhibit their transfer to the aboveground parts, thus reducing the distribution ratio of heavy metals to the aboveground parts, reducing the toxic effect of heavy metals on important aboveground physiological organs of plants, and making plants have a higher tolerance to Mn stress.

### 4.4 The effects of AMF on subcellular components of Mn under Mn stress

Compartmentalization is a crucial strategy for detoxifying heavy metals in plant cells because after entering into plant cells, heavy metals can bind to different subcellular compartments (e.g., cell wall and soluble fraction), exhibiting different ecotoxicological significance. The present study showed that Mn in the roots, stems, and leaves of *L. davidii* is mainly distributed in the cell wall and soluble components. Compared with CK, the proportions of Mn in the cell wall and soluble components of the roots, stems, and leaves of *L. davidii* were higher, while the proportion of chloroplast and nuclear components, and mitochondrial components were lower. This showed that the cell wall components and soluble components are the main storage sites of Mn in various organs of *L. davidii*, and most of the Mn in *L. davidii* is fixed in the cell wall or isolated in vacuoles (the soluble fraction mainly exists in vacuoles). It is usually known that heavy metals are less toxic to plant cells when they are distributed in cell walls and vacuoles (the soluble fraction) but more toxic when they are distributed in chloroplasts, nuclei, mitochondria, and other organelles. Therefore, it is one of the growth strategies for tolerant plants to adapt to heavy metal stress environment to combine toxic or excessive heavy metal ions on plant cell walls or promote their separation in vacuoles, thus reducing the toxicity of Mn to organelles, which is one of the mechanisms of *L. davidii* for detoxifying Mn.

In addition, compared with that in the uninoculated group, it was found that the proportion of Mn content in the cell wall components and the soluble components in leaves in the inoculated group increased. This indicated that AMF enhanced the cell wall fixation and vacuole compartmentalization (the soluble fraction mainly composed in vacuoles) of *L. davidii* under Mn stress and reduced the toxicity of Mn to the organelles of *L. davidii*. Similarly, under cadmium stress, most cadmium (more than 90%) accumulated in the cell wall and soluble parts in *Zea mays*, while a small amount of cadmium exists in organelles, and AMF symbiosis promoted cadmium transfer to vacuoles (Zhang et al., 2019). Therefore, cell wall components may be the main site for Mn fixation in roots, which can reduce the transport of cadmium from roots to the upper ground (Gao et al., 2021).

### 4.5 The effects of AMF on chemical forms of Mn under Mn stress

The chemical forms of heavy metals are a major determinant of physiological functions and toxicity; different forms of heavy metals have different bioavailability and toxicity. Among them, ethanol and deionized water have the highest activity and mobility in the extracted state, which are the easiest to enter the plant symplast, and have the strongest toxicity to plants. The heavy metals extracted from sodium chloride (NaCl) are mainly combined with pectin and protein, and the solubility of heavy metals extracted from acetic acid (HAc) and hydrochloric acid (HCl) is low. The activity and mobility of these extracted heavy metals are low, and their toxicity to plants is weak. The residue state is the most stable, and the toxicity to plants is the most weak. The present study showed that, with the increasing Mn concentration, the content of different chemical forms of Mn in the stems of *L. davidii* in the uninoculated group and the inoculated group showed an increasing trend, and the Mn in the sodium chloride extract, acetic acid extract, and hydrochloric acid extract of *L. davidii* was more than that in the ethanol extract and deionized water extract. This illustrated that Mn stress made the activity and mobility of Mn ions in *L. davidii* lower, thus reducing the toxicity of Mn stress to *L. davidii*.

In addition, the present study showed that the Mn in *L. davidii* in the inoculated group had a higher ratio when extracted using sodium chloride, acetic acid, and hydrochloric acid than that in the uninoculated group. After inoculation with AMF, the proportion of sodium chloride extracted, acetic acid extracted, and residual Mn in *L. davidii* increased significantly, while the proportion of ethanol extracted and deionized water extracted Mn decreased significantly. This showed that AMF promoted the transformation of Mn ions in *L. davidii* from ethanol and deionized water extracts with strong activity to sodium chloride and acetic acid extracts with weak activity, thus reducing the damage of Mn ions to the cells and structures of *L. davidii*, maintaining its normal physiological and biochemical activities, and enhancing the Mn tolerance of *L. davidii*. Similarly, after inoculation with AMF, Li et al. (2016) also found that AMF may augment the tolerance to Cd of rice by decreasing the proportions of Cd in active forms (FE and FW) in 0.05–0.1 mM Cd solutions. AMF inoculation under cadmium stress increased the proportion of cadmium with weak active state in *M. sativa* (Wang et al., 2012). Therefore, AMF may also improve the resistance to Mn of *L. davidii* by converting Mn into inactive forms that are less toxic.

## 5 Conclusion

This study reports the effects of AMF inoculation and Mn stress on plant growth, physiological and biochemical characteristics, Mn uptake, subcellular distribution, and chemical forms of Mn in *L. davidii*. The results showed that the biomass of the AMF-inoculated plants was higher than that of the uninoculated plants, and the content of MDA in the inoculated plants was lower than that in the uninoculated plants, which showed that AMF can alleviate Mn toxicity. AMF inoculation enhanced the enrichment of Mn in *L. davidii* and enhanced the fixation of Mn in *L. davidii* roots so as to alleviate the damage of Mn stress to its aboveground parts. At low Mn substrate concentrations, the cell wall appears to play an important role in Mn retention, while the main subcellular fraction that contributed to Mn detoxification is the vacuoles at high Mn substrate concentrations (≥10 mM). Furthermore, the concentrations and proportions of Mn extracted by ethanol and d-H_2_O were also lower in the stems, and roots of the inoculated plants compared with the uninoculated plants at high substrate concentrations (≥10 mM), indicating that AMF can convert Mn into inactive forms, which are less toxic. The results of this study illustrated that inoculation with AMF could adjust the osmotic regulation system and antioxidant system to stabilize the physiological environment and accumulate more Mn in cell walls and soluble fractions to improve the tolerance of *L. davidii* to manganese stress.

## Data Availability

The original contributions presented in the study are included in the article/**Supplementary Material**. Further inquiries can be directed to the corresponding authors.

## Supplementary material

Supplementary Figure S1 Effect of AMF on Mn content in subcellular fractions of *L. davidii* root under Mn stress. Note: F1, cell wall fraction; F2, chloroplasts and cell nuclei fraction; F3, mitochondria fraction; F4, ribosomes fraction.

Supplementary Figure S2 Effect of AMF on Mn content in subcellular fractions of *L. davidii* stems under Mn stress. Note: F1, cell wall fraction; F2, chloroplasts and cell nuclei fraction; F3, mitochondria fraction; F4, ribosomes fraction.

Supplementary Figure S3 Effect of AMF on Mn content in subcellular fractions of *L. davidii* leaves under Mn stress.Note: F1, cell wall fraction; F2, chloroplasts and cell nuclei fraction; F3, mitochondria fraction; F4, ribosomes fraction.

Supplementary Figure S4 Effect of AMF on manganese chemical forms of manganese of *L. davidii* root under Mn stress. Note: F1, 80% ethanol; F2, deionized H_2_O; F3, 1 M NaCl; F4, 2% HA; F5, 0.6 M HCl; F6, residue.

Supplementary Figure S5 Effect of AMF on manganese chemical forms of manganese of *L. davidii* stems under Mn stress. Note: F1, 80% ethanol; F2, deionized H_2_O; F3, 1 M NaCl; F4, 2% HA; F5, 0.6 M HCl; F6, residue.

Supplementary Figure S6 Effect of AMF on manganese chemical forms of manganese of *L. davidii* leaves under Mn stress. Note: F1, 80% ethanol; F2, deionized H_2_O; F3, 1 M NaCl; F4, 2% HA; F5, 0.6 M HCl; F6, residue.
