# Supplementary figures and images for: Arbuscular mycorrhizal fungi alleviate Mn phytotoxicity by altering Mn subcellular distribution and chemical forms in Lespedeza davidii
Source: Front Plant Sci. 2024 Nov 27;15:1470063. doi: 10.3389/fpls.2024.1470063 (PMC11631616; doi:10.3389/fpls.2024.1470063)

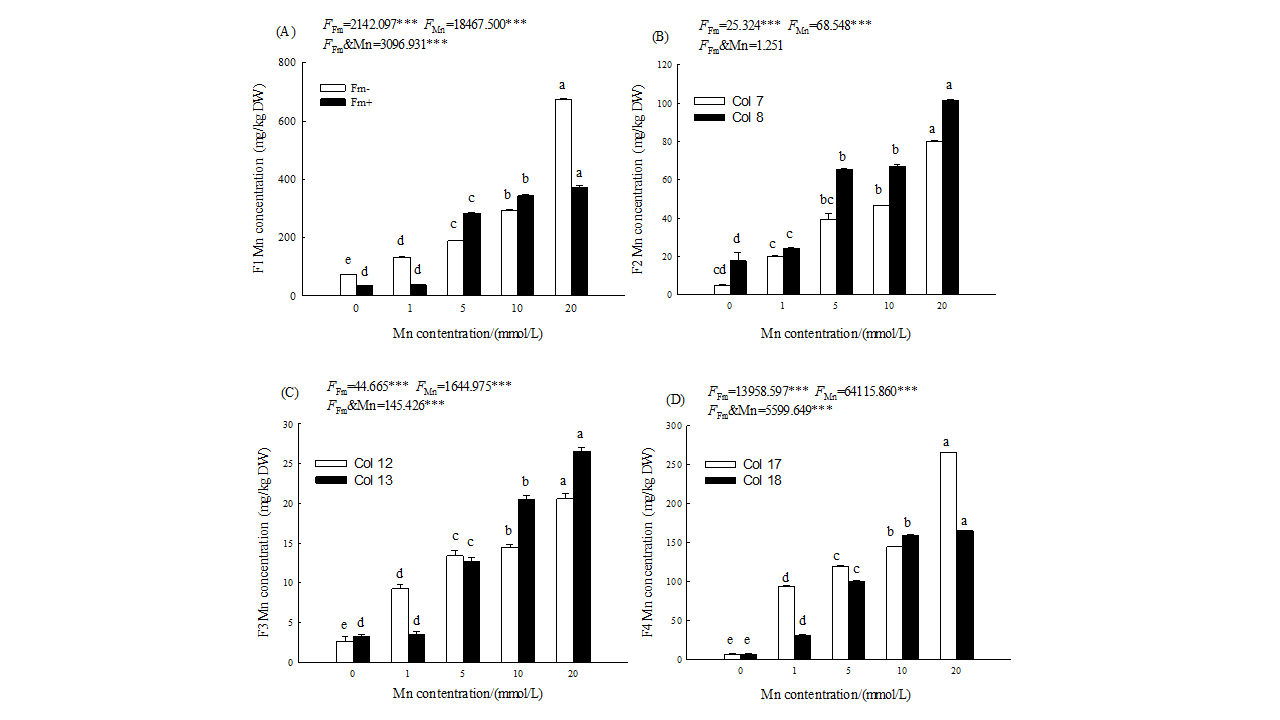

Supplement: Supplementary Figure S1 — Effect of AMF on Mn content in subcellular fractions of L. davidii root under Mn stress. Note: F1, cell wall fraction; F2, chloroplasts and cell nuclei fraction; F3, mitochondria fraction; F4, ribosomes fraction. [file Image1.tif]

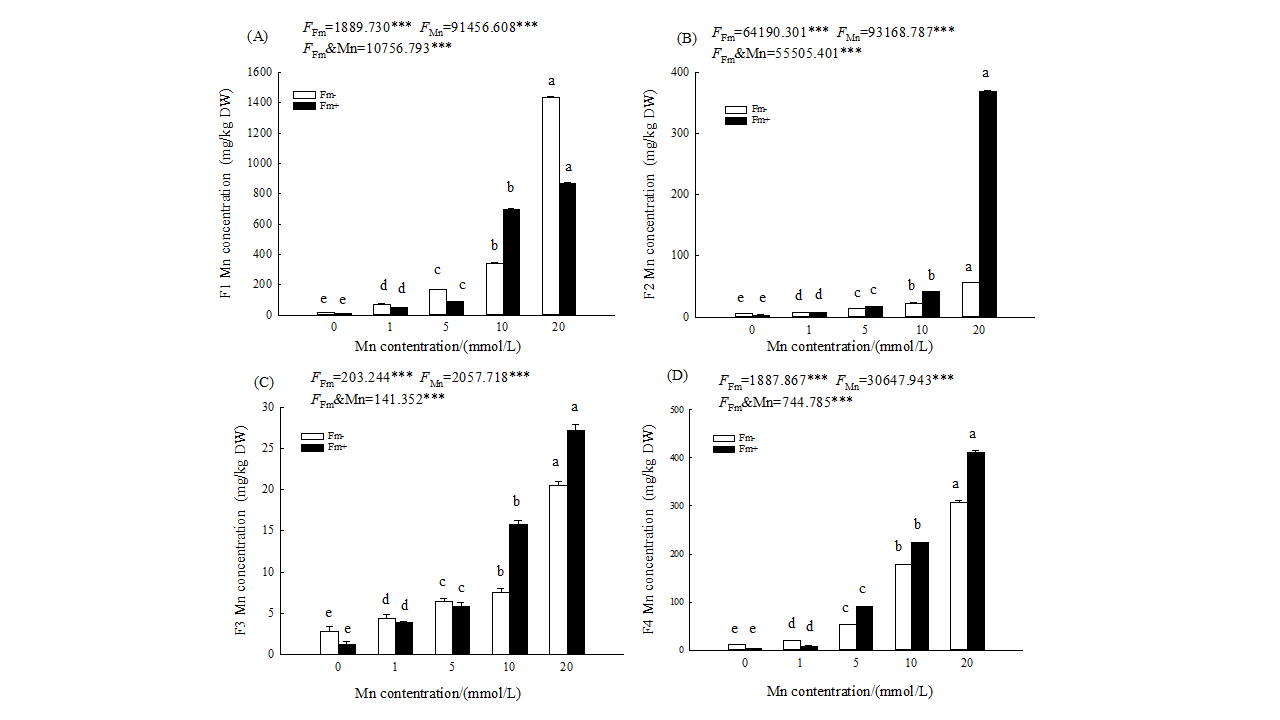

Supplement: Supplementary Figure S2 — Effect of AMF on Mn content in subcellular fractions of L. davidii stems under Mn stress. Note: F1, cell wall fraction; F2, chloroplasts and cell nuclei fraction; F3, mitochondria fraction; F4, ribosomes fraction. [file Image2.tif]

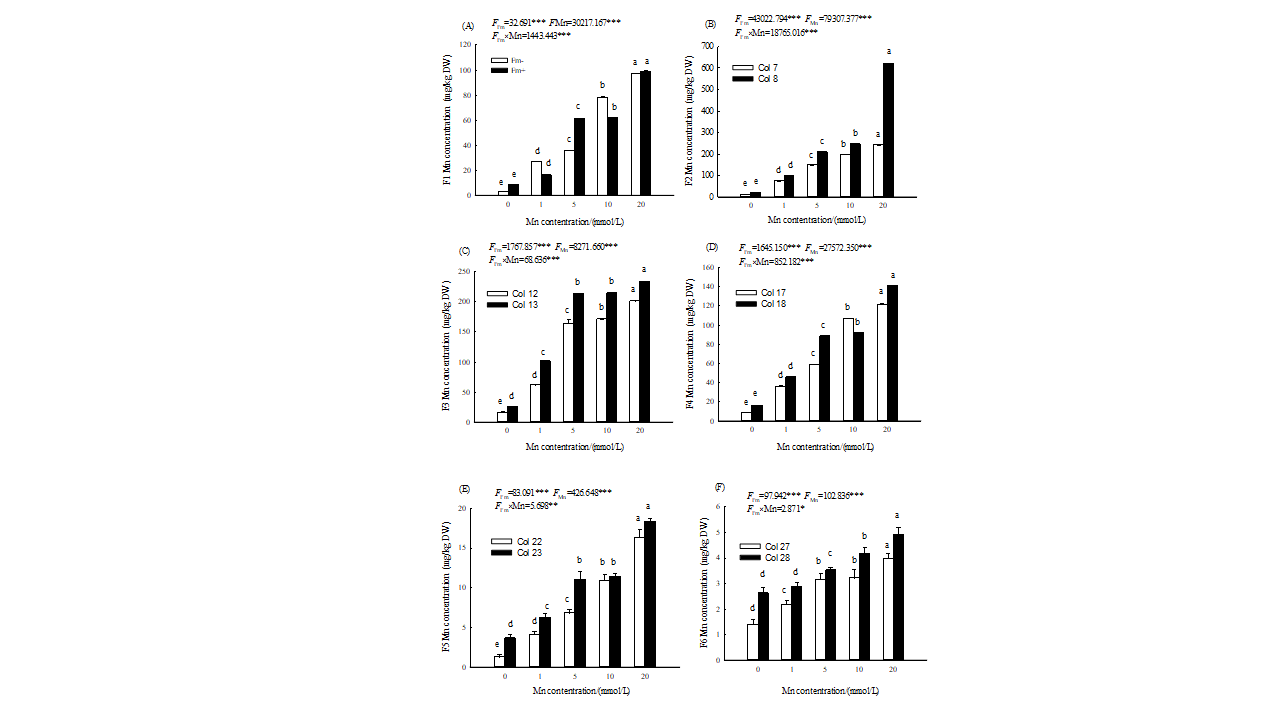

Supplement: Supplementary Figure S4 — Effect of AMF on manganese chemical forms of manganese of L. davidii root under Mn stress. Note: F1, 80% ethanol; F2, deionized H2O; F3, 1 M NaCl; F4, 2% HA; F5, 0.6 M HCl; F6, residue. [file Image4.tif]

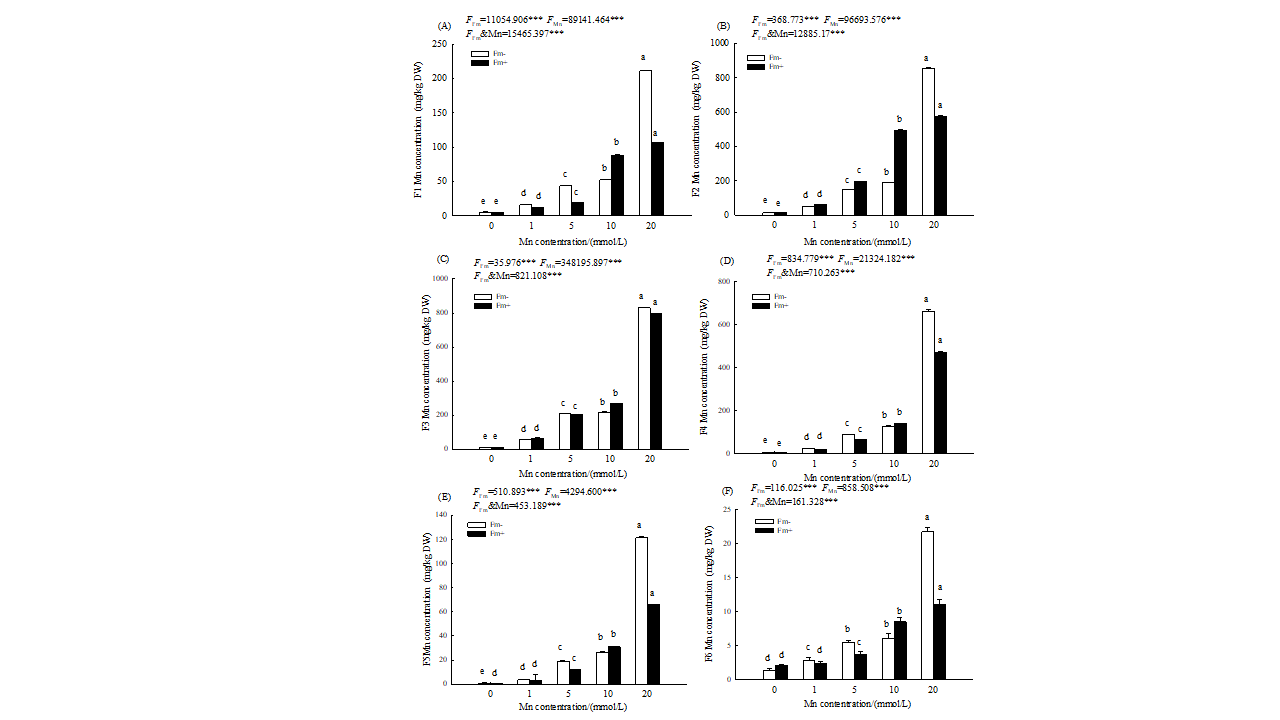

Supplement: Supplementary Figure S5 — Effect of AMF on manganese chemical forms of manganese of L. davidii stems under Mn stress. Note: F1, 80% ethanol; F2, deionized H2O; F3, 1 M NaCl; F4, 2% HA; F5, 0.6 M HCl; F6, residue. [file Image5.tif]

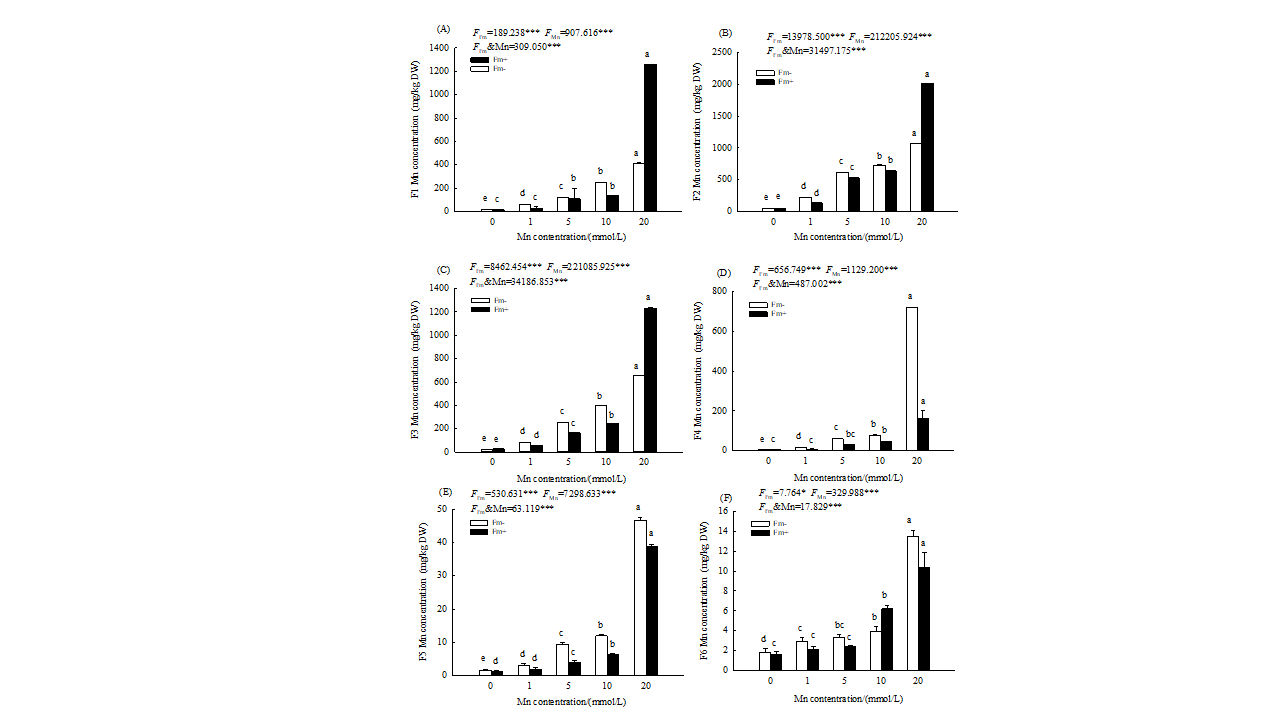

Supplement: Supplementary Figure S6 — Effect of AMF on manganese chemical forms of manganese of L. davidii leaves under Mn stress. Note: F1, 80% ethanol; F2, deionized H2O; F3, 1 M NaCl; F4, 2% HA; F5, 0.6 M HCl; F6, residue. [file Image6.tif]
